# The Stability and Activity Changes of Apigenin and Luteolin in Human Cervical Cancer Hela Cells in Response to Heat Treatment and Fe^2+^/Cu^2+^ Addition

**DOI:** 10.3390/foods8080346

**Published:** 2019-08-14

**Authors:** Wan-Ning Liu, Jia Shi, Yu Fu, Xin-Huai Zhao

**Affiliations:** 1 Key Laboratory of Dairy Science, Ministry of Education, Northeast Agricultural University, Harbin 150030, Heilongjiang, China; 2 College of Food Science, Southwest University, Chongqing 400715, China

**Keywords:** apigenin, luteolin, degradation, ferrous ions, cupric ions, cervical cancer cells, growth inhibition, apoptosis

## Abstract

Flavonoids are natural polyphenolic compounds with desired bio-functions but with chemical instability and sensitivity to temperature, oxygen, and other factors. Apigenin and luteolin, two flavones of the flavonoid family in plant foods, were; thus, assessed and compared for their stability, especially the changes in anti-cancer activity in response to the conducted heat treatments and the addition of ferrous or cupric ions. The two flavones in aqueous solutions showed first-order degradation at 20 and 37 °C. The addition of ferrous or cupric ions (except for Cu^2+^ at 37 °C) enhanced luteolin stability via forming the luteolin–metal complexes; however, Fe/Cu addition (especially at 37 °C) consistently impaired apigenin stability. Using the human cervical cancer Hela cells and two cell treatment times (24 and 48 h), it was evident that heat treatments (37 and 100 °C) or Fe/Cu addition could endow apigenin and luteolin with decreased activities in growth inhibition, DNA damage, intracellular reactive oxygen species (ROS) generation, and apoptosis induction. In general, higher temperature led to greater decrease in these activities, while Fe^2+^ was more effective than Cu^2+^ to decrease these activities. The correlation analysis also suggested that the decreased ROS generation of the two flavones in the Hela cells was positively correlated with their decreased apoptosis induction. It is; thus, concluded that the two treatments can influence the two flavones’ stability and especially exert an adverse impact on their anti-cancer activities.

## 1. Introduction

Flavonoids are a class of secondary plant phenolic compounds existing in a wide range of human diets. Flavonoids are interesting target compounds to many researchers because they have anti-oxidative, anti-microbial, anti-inflammatory, and anti-cancer effects [1]. Flavonoids, as natural anti-oxidants, even can exert stronger anti-oxidant activity than that of anti-oxidative vitamins and synthetic phenols [2]. Flavonoid compounds, such as hesperetin, naringenin, poncirin, and diosmetin, are effective to inhibit harmful microorganisms; for example, they can inhibit the growth of *Helicobacter pylori* [3]. Furthermore, flavonoids have profound immune-regulatory and anti-inflammatory effects [4]. Cocoa flavonoids had immuno-regulation in the EL4.BU.OU6 cells by increasing the release of interleukin-4 [5]. Rutin, hesperidin, hesperetin, and quercetin were effective for both chronic and neurogenic inflammation [6]. Moreover, many researchers have paid special attention to the anti-cancer functions of flavonoids and flavonoid extracts. Quercetin, luteolin, chrysin, kaempferol, apigenin, and myricetin have cytotoxic effects on the human esophageal adenocarcinoma OE33 cells, resulting in growth inhibition, cell-cycle arrest, and apoptosis [7]. Baicalin could inhibit the growth of several human prostate cancer cells, including DU145, PC-3, LNCaP, and CA-HPV-10 cells [8]. Naringenin from citrus fruits could inhibit the proliferation of human colon cancer HT29 cells [9]. All results suggest that dietary flavonoids are promising natural compounds with desired ability to reduce cancer risk. Subsequently, an inverse correlation between flavonoid intake and the incidence of laryngeal and esophageal cancers has been reported [10].

Fe and Cu are two essential trace elements in the body, and are widely found in human diets. Fe/Cu ions have active redox property and thus can easily react with dietary flavonoids, which might alter chemical structures, especially the bio-functions of flavonoids. When flavonoids interact with Fe/Cu ions, they are oxidized by the two ions with decreased absorbance at their maximum absorption peaks [11]. Flavonoids can chelate with the two ions and thus form complexes with changed properties. Flavonoid–Fe^2+^ complexes showed enhanced stability, while flavonoid–Cu^2+^ complexes had auto-oxidation [12,13]. Furthermore, flavonoid oxidation by Cu^2+^ was irreversible [13]. However, superoxide scavenging capacities of rutin, taxifolin, epicatechin, and luteolin were weaker than their respective Fe/Cu complexes [14]. Overall, it is reasonable to believe that the anti-cancer potentials of flavonoids could be affected by these transition metal ions.

During food processing, Fe/Cu ions may easily enter food matrices, as food matrices have the opportunity to contact the surfaces of pipes and equipment made from the two metals. Furthermore, some treatments used in food processing might exert potential impacts on dietary flavonoids; for example, heat treatment is necessary or unavoidable. In general, flavonoids are sensitive to high temperature [15], because high temperature can promote their degradation. The higher temperature of elderberry anthocyanins gave rise to higher degradation rate constants [16], while flavonoids in cloudy apple juice at 80 to 145 °C also experienced increased degradation rates [17]. Dietary flavonoids at higher temperatures; therefore, might be endowed with changed bio-functions, mainly due to flavonoid degradation. Brazilian bean after boiling and draining had decreased flavonoid content and lower anti-oxidant capacity [18]. Thermal treatment of galangin, kaempferol, morin, and myricetin led to weakened growth inhibition on the human colon carcinoma HCT-116 cells [19,20]. Thus, the effects of heat treatment and metal entrance on anti-cancer functions of flavonoids in other cancer cells, like the human cervical cancer Hela cells, deserve further study.

**Figure 1:**
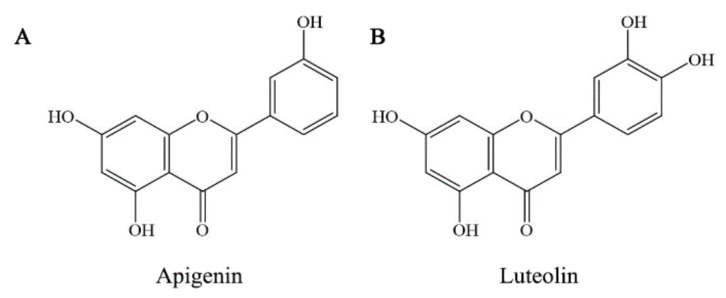
The chemical structures of flavone compounds apigenin and luteolin.

The flavones are commonly found flavonoid compounds in natural foods, among those flavone members are apigenin and luteolin. Apigenin is rich in Chinese cabbage, bell pepper, garlic, bilimbi fruit, guava, wolfberry leaves, and local celery, while luteolin is rich in bird chili, onion leaves, and bilimbi fruit and its leaves [21]. Normally, flavones had been reported to have stronger anti-cancer activities due to their high lipophilicity [22]. Apigenin is a promising anti-cancer compound, because it could inhibit the growth of several cancer cells [23]. Luteolin also is served as a potential and emerging anti-cancer compound, due to its clear toxic effect on eukaryotic DNA topoisomerase I [24]. From a chemical point of view, apigenin and luteolin have several −OH groups in their molecules (Figure 1) and thus have different stability once they are heated or mixed with Fe/Cu ions. Whether apigenin and luteolin after these treatments still have good anti-cancer functions is important but unsolved at present. Such a study; thus, deserves consideration.

In this study, both apigenin and luteolin were measured for their stability under two temperatures (20 and 37 °C) or Fe^2+^/Cu^2+^ addition. The two temperatures are regarded as room temperature of diet storage and average temperature of the body, respectively. Moreover, the latter is also the culture temperature of most cells. Afterwards, the two flavones were subjected to heat treatments at 37 and 100 °C or Fe/Cu addition, and then evaluated for their changes in anti-cancer activity using the human cervical cancer Hela cells as a cell model. Four indices including growth inhibition, cell morphology (or DNA damage), reactive oxygen species (ROS) generation, and apoptosis induction were used to clarify or compare activity changes. The study aimed to reveal whether the two treatments (heat treatment and Fe/Cu addition) could affect the stability of apigenin and luteolin as well as their anti-cancer effects in Hela cells.

## 2. Materials and Methods

### 2.1. Chemicals and Reagents

The apigenin and luteolin (purity >98%) were bought from Dalian Meilun Biological Technology Co. Ltd. (Dalian, Liaoning, China). The cell counting kit-8 (CCK-8) was purchased from Dojindo Molecular Technologies Inc. (Kyushu, Japan). The reactive oxygen species (ROS) assay kit, Annexin V-FITC apoptosis detection kit, and Hoechst 33258 kit were obtained from Beyotime Institute of Biotechnology (Shanghai, China). 5-Fluorouracil (5-Fu) was bought from Tianjin Jinyao Pharmaceutical Co. Ltd. (Tianjin, China). All other chemicals used were of analytical grade. The water used in this study was ultrapure water generated with Milli-Q PLUS (Millipore Corporation, New York, NY, USA).

### 2.2. Cell Line and Culture Conditions

The Hela cells (STR: Amelogenin: X; CSF1PO: 9,10; D13S317: 12,13.3; D16S539: 9,10; D18S51: 16; D19S433: 13, 14; D21S11: 27,28; D2S1338: 17; D3S1358: 15, 18; D5S818: 11, 12; D7S820: 8,12; D8S1179: 12, 13; FGA: 18,21; TH01: 7; TPOX: 8,12; vWA: 16,18) used in this study were purchased from the Cell Bank of Shanghai Institute of Biochemistry and Cell Biology (Shanghai, China). As recommended by the cell supplier, the cells were cultured in the Dulbecco’s modified eagle’s medium (DMEM) (Sigma-Aldrich, Co. St. Louis, MO, USA) supplemented with 10% fetal bovine serum (FBS) (Hyclone, Logan, UT, USA) and 1% penicillin/streptomycin solution at 37 °C in a 5% CO_2_ atmosphere.

### 2.3. Assays of Degradation Rates of the Two Flavones

Both apigenin and luteolin were dissolved in dimethyl sulfoxide (DMSO) to prepare stock solutions of 0.1 mol/L. The stock solutions were diluted with ethanol and then with 0.1 mol/L phosphate buffer solution (PBS, pH 7.3) to two final concentrations of 20 and 30 μmol/L, using respective dilution factors of 5000 and 3333. Otherwise, the stock solutions were diluted with ethanol and PBS similarly but with addition of CuCl_2_ or FeCl_2_, which resulted in a fixed molar ratio of flavones to Fe/Cu (3:1). All prepared solutions were incubated at two temperatures (20 and 37 °C) for 6 h, and measured for their absorbance values at various time points using two wavelengths (apigenin 354 nm; luteolin 360 nm) and a UV-visible spectrophotometer (UV-2401 PC, Shimadzu Co., Kyoto, Japan). PBS was used as blank in this assay. Residual levels of apigenin and luteolin were estimated using the respective standard curves generated from a serial of standard solutions.

Based on the established first-order reaction model of flavonoid degradation [25], the degradation rate constants (k, h^−1^) of apigenin and luteolin were calculated using a derived linear regression equation.

### 2.4. Treatments of the Two Flavones for Cell Experiments

Apigenin and luteolin were dissolved in DMSO to obtain 0.3 moL/L stock solutions, and diluted by the DMEM supplemented with 5% FBS to yield flavone concentrations of 20 to 80 μmoL/L using the dilution factors ranging from 15,000 to 3750. The stock solutions were also diluted by DMEM without FBS to a fixed flavone concentration of 42.1 μmoL/L (using dilution factor of 7126), and heated in the dark with a thermostatic water bath operated at 37 °C (or 100 °C) for 6 h (or 0.5 h). After heat treatment, the two solutions were immediately cooled in the ice water and added with the FBS to yield a final flavone concentration of 40 μmoL/L. The FBS was not involved in these thermal treatments. Or else, the stock solutions were diluted with DMEM supplemented with 5% FBS, and added with 100 mmoL/L CuCl_2_ or FeCl_2_ solution to yield a final flavone concentration (40 μmoL/L) together with a fixed molar ratio (3:1) of flavones to Fe/Cu.

### 2.5. Assay of Growth Inhibition

The cells (1 × 10^4^ cells per 100 μL per well) were seeded onto the 96-well plates and incubated for 12 h. After removal of cell medium, the cells were treated with 0.1% DMSO (negative control), 100 μmol/L 5-Fu (positive control), and the prepared flavone samples for 24 and 48 h, respectively, and then washed twice with the PBS. The CCK-8 solution of 100 μL (10 μL CCK-8 plus 90 μL DMEM containing 5% FBS) was added to each well, and the cells were further incubated at 37 °C for 1.5 h. A microplate reader (Bio-Rad Laboratories, Hercules, CA, USA) was then used to measure the optical density values at 450 nm, which were used to calculate the percentages of growth inhibition as previously described [26].

### 2.6. Hoechst 33258 Staining

The cells in 6-well plates were grown to 70% confluence and incubated with the untreated or treated flavone samples (40 μmol/L) for 24 h. After discarding cell media, 4% methanol of 1 mL was added into each well to fix the cells at 4 °C for 10 min. After washing twice with the PBS buffer, the Hoechst 33258 (200 mg/mL) of 1 mL was added into each well to stain the cells for 10 min. The cells were then observed under a fluorescence microscope (Zeiss Axio Observer A1m, Carl Zeiss, Oberkochen, Germany), while cell images were taken at 350 nm using an objective of 40-fold.

### 2.7. Assay of Apoptosis Induction

The proportions of the apoptotic cells in different cell groups were detected using flow cytometry technique and Annexin V-fluorescein isothiocyanate (FITC)/propidium iodide (PI) double staining as previously described [27]. The cells were grown to 70% confluence in 6-well plates, incubated with the untreated and treated flavones at 40 μmoL/L for 24 and 48 h, harvested, washed with the cold PBS, and centrifuged at 110× *g* for 5 min to discard the supernatants. The pellets were re-suspended gently in the Annexin V-FITC binding buffer of 200 μL, and incubated with the Annexin V-FITC of 10 μL for 15 min in the dark at 20 °C. The binding buffer (300 μL) and PI (5 μL) were added into each well and mixed gently. The stained cells were assayed with a flow cytometer (FACS Calibur, Becton Dickson, San Jose, CA, USA), to detect the percentages of necrotic, late apoptotic, intact, and early apoptotic cells (Q1–Q4).

### 2.8. Assay of Intracellular Reactive Oxygen Species

In this assay, the cells were treated similarly as those in the assay of apoptosis induction. After cell harvesting and PBS washing, the cells were re-incubated with 20,70-dichlorofluorescein (DCF-DA, 5 mmoL/L) at 37 °C for 30 min in the dark, washed three times with the PBS, and re-suspended in the PBS of 1 mL. The cell suspension was seeded onto the 96-well plates and measured for their fluorescence intensities using a fluorescence microplate reader (Infinite 200, Tecan, Männedorf, Switzerland) and respective emission and excitation wavelengths of 488 and 525 nm. The relative ROS levels were expressed as the percentages of the control cells as previously described [28].

### 2.9. Statistical Analysis

All reported data collected from three independent experiments or assays were expressed as means or means ± standard deviations, and compared using the SPSS 20.0 software (SPSS Inc., Chicago, IL, USA). All obtained data meet the assumptions of normality and constant variance. Significant differences (*p* < 0.05) between the means of multiple groups were evaluated by the one-way analysis of variance with Duncan’s multiple range tests and two-way analysis of variance (ANOVA). The Pearson’s correlation coefficient was also calculated using this software.

## 3. Results

### 3.1. Instability of Apigenin and Luteolin at Two Temperatures or in the Presence of Fe^2+^/Cu^2+^

**Figure 2:**
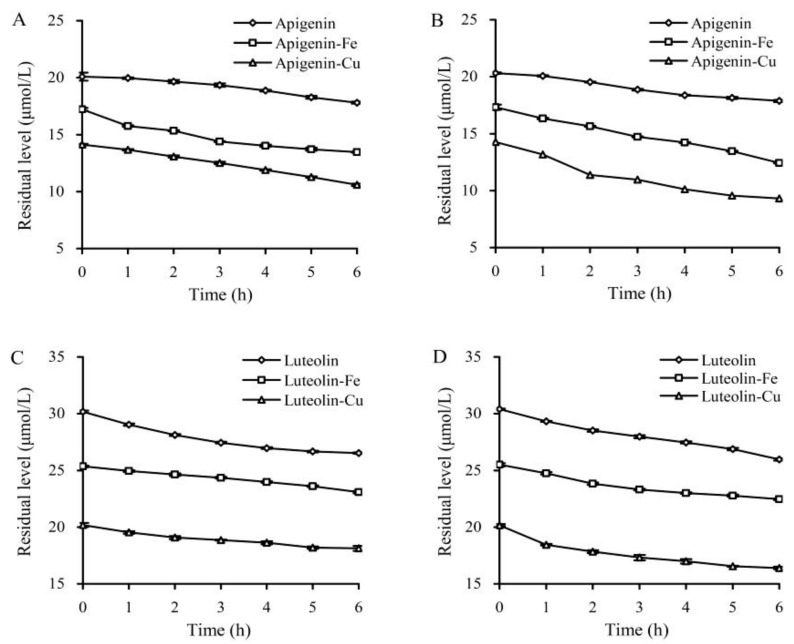
Residual levels of apigenin and luteolin in the solutions incubated at 20 °C (**A**,**C**) and 37 °C (**B**,**D**) for different time periods.

Apigenin and luteolin showed typical UV-spectra with maximum absorption peaks around 354 and 360 nm, respectively. This study; thus, used two wavelengths to detect residual apigenin and luteolin, which were exposed to two temperatures or Fe^2+^/Cu^2+^ for different time periods. The results indicated that both apigenin and luteolin were chemically instable in these cases, because their residual levels showed a decreasing trend (Figure 2). The calculated degradation rate constants (*k*) revealed how the higher temperature (37 °C) and the two ions affected the stability of apigenin and luteolin (Table 1). When their solutions were kept at 20 or 37 °C, apigenin and luteolin showed *k* values of 0.0207 and 0.0214 or 0.0226 and 0.0245 h^−1^, respectively. Higher temperature clearly led to higher *k* value (i.e., decreased stability). In the presence of Fe^2+^/Cu^2+^, apigenin gave greater degradation, because the measured *k* values were increased to 0.0395–0.0728 h^−1^. More importantly, higher temperature (37 °C) combined with Cu^2+^ brought about more drastic apigenin degradation. As for luteolin, Fe^2+^ resulted in lower *k* values (i.e., decreased degradation), while Cu^2+^ at 37 °C led to enhanced degradation (i.e., larger *k* value). These results suggested that: (1) Both higher temperature and Fe^2+^/Cu^2+^ caused structural instability for apigenin; and (2) only higher temperature and Cu^2+^ could increase the instability of luteolin. Both heat treatments and Cu/Fe addition; therefore, might alter the anti-cancer activities of these two flavones.

### 3.2. Growth Inhibition of the Flavone Samples on Hela Cells

**Figure 3:**
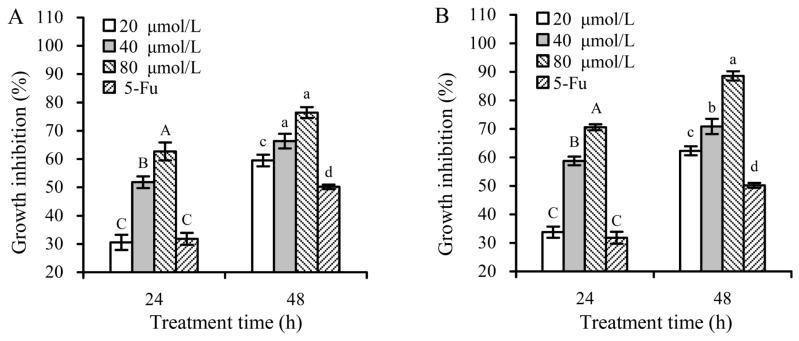
Growth inhibition of apigenin (**A**) and luteolin (**B**) of various concentrations on the Hela cells at treatment times of 24 and 48 h. 5-Fu, 5-fluorouracil as a positive control. Different capital or lowercase letters above the columns indicate that the means within the same group differ significantly according to one-way ANOVA (*p* < 0.05).

The CCK-8 assaying results indicated that both apigenin and luteolin at 20–80 μmoL/L dose- and time-dependently had cytotoxic effects on the Hela cells (Figure 3), resulting in inhibition percentages of 30.6%–62.7% and 33.8%–70.6% (24 h) or 59.5%–76.4% and 62.3%–88.6% (48 h), respectively. Both apigenin and luteolin at 40 μmoL/L caused corresponding inhibition percentages of 52.0% and 57.9% (24 h) or 65.7% and 73.2% (48 h) in the cells. Thus, flavone concentration of 40 μmol/L was used in later study, because this concentration led to growth inhibition up to 50%–70%.

**Figure 4:**
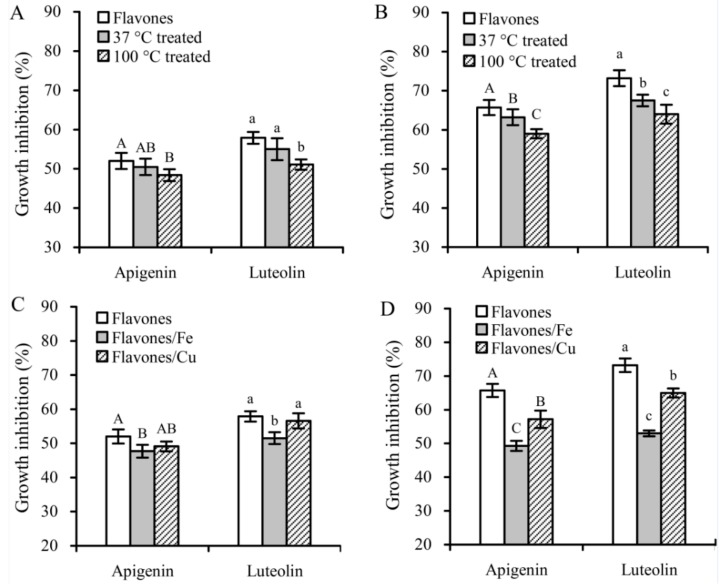
Growth inhibition of 40 μmoL/L flavonols (with or without thermal treatments and Fe/Cu addition) on the Hela cells with treatment times of 24 (**A**,**C**) and 48 h (**B**,**D**). Different capital or lowercase letters above the columns indicate that the means within the same group differ significantly according to one-way ANOVA (*p* < 0.05).

This flavone concentration was then used to compare different growth inhibition of these flavone samples with or without heat treatment or Fe/Cu addition in the Hela cells (Figure 4). Heat treatment at 37 °C decreased the inhibition percentages of apigenin and luteolin to 50.5% and 55.0% (24 h) or 63.2% and 67.5% (48 h), respectively. Heat treatment at 100 °C brought about much decreased growth inhibition, because the measured inhibition percentages of apigenin and luteolin were reduced to 48.4% and 51.1% (24 h) or 59.0% and 64.0% (48 h), respectively. Overall, heat treatment at 100 °C and Fe addition showed greater potential to decrease growth inhibition of the two flavones.

### 3.3. Morphological Alteration of Hela Cells Treated by the Flavone Samples

**Figure 5:**
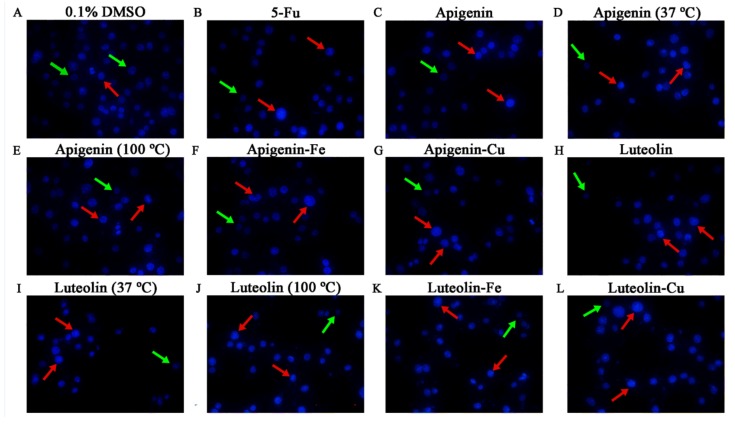
Morphological features of the Hela cells treated with 0.1% DMSO, 100 μmol/L 5-fluorouracil (5-Fu), and 40 µmol/L flavone samples (with or without thermal treatment and Fe/Cu addition) for 24 h. A fluorescence microscope was used to photograph images (40×). The red and green arrows indicate the corresponding apoptotic and intact cells.

Morphological alteration of the treated cells can reflect potential apoptosis induction of the target substances. Morphological features of the treated Hela cells were; thus, observed using the Hoechst 33258 staining and fluorescence microscopy. In these results, the cell nuclei were dyed and observed in the fluorescent images. The apoptotic cells were observed as light blue while the viable cells were observed as dark blue. Moreover, the apoptotic cells often showed apoptotic morphology as the condensation and fragmentation of nuclei shrinkage as well as the formation of apoptotic bodies. In general, the untreated flavones were more effective than the treated ones to alter the morphological features of Hela cells, while 100 °C treatment and Fe addition brought about relatively higher cell density (Figure 5). Compared with the control cells without any treatment, the treated cells showed the typical apoptotic morphology and decreased cell density in the observation field. These results suggested that these assessed samples could damage DNA and thus had potential (but different) apoptosis induction towards the Hela cells.

### 3.4. Pro-Oxidation of the Flavone Samples

The Hela cells treated with or without these flavone samples were; thus, detected for their ROS levels (Table 2). The results indicated that all assessed samples had pro-oxidation in the cells, as the treated cells showed increased relative ROS levels (larger than 200%) than in the control cells (*p* < 0.05). The untreated apigenin and luteolin brought about relative ROS levels of 229% and 284% (24 h) or 263% and 281% (48 h), respectively. The apigenin and luteolin treated at 37 °C for 6 h resulted in lower ROS levels of 212% and 272% (24 h) or 260% and 263% (48 h), respectively. Apigenin and luteolin treated at 100 °C for 0.5 h showed much weaker ability to increase ROS generation than those heated at 37 °C for 6 h. For apigenin and luteolin, Fe addition led to the highest ROS reduction in the cells; however, Cu addition only decreased ROS levels to a slight extent, compared with Fe addition. Overall, both heat treatment and Fe/Cu addition consistently led to decreased ROS generation in the Hela cells.

However, ROS generation of luteolin at 48 h was lower than that at 24 h (except 100 °C heat treatment) (Table 2). In these cases, the respective samples had stronger pro-oxidation, could enhance ROS to much higher levels and, thereby, caused greater cell apoptosis, which led to a lower number of viable cells. After a longer period, only a few viable cells continued to generate ROS. Finally, ROS generation with incubation time of 48 h was less than that with incubation time of 24 h.

### 3.5. Apoptosis Induction of the Flavone Samples

**Figure 6:**
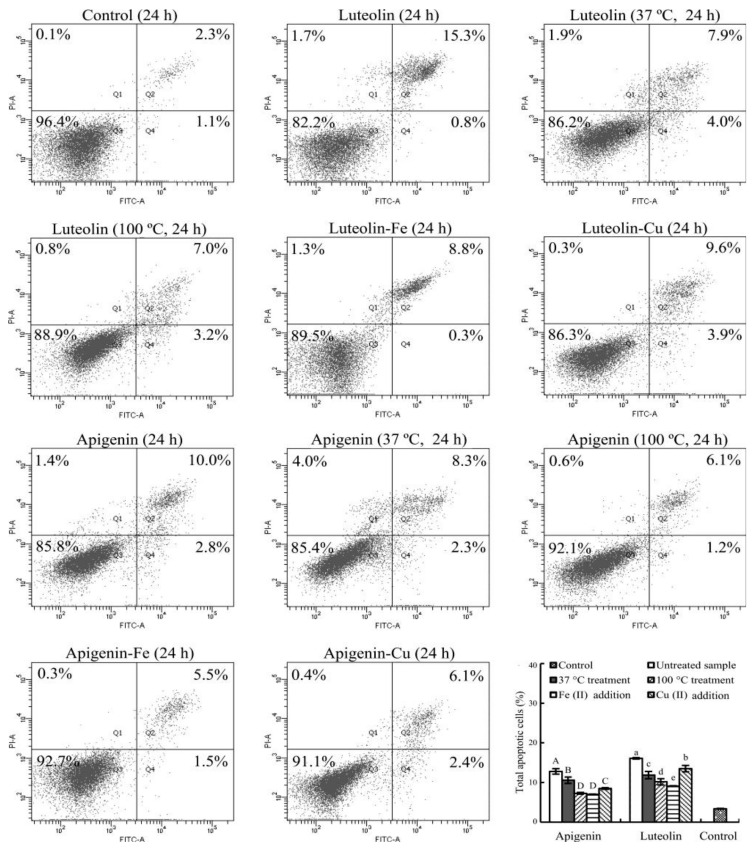
Cell percentages of the Hela cells treated with 0.1% DMSO (control) and 40 µmoL/L flavone samples with or without thermal treatments and Fe/Cu addition for 24 h.

**Figure 7:**
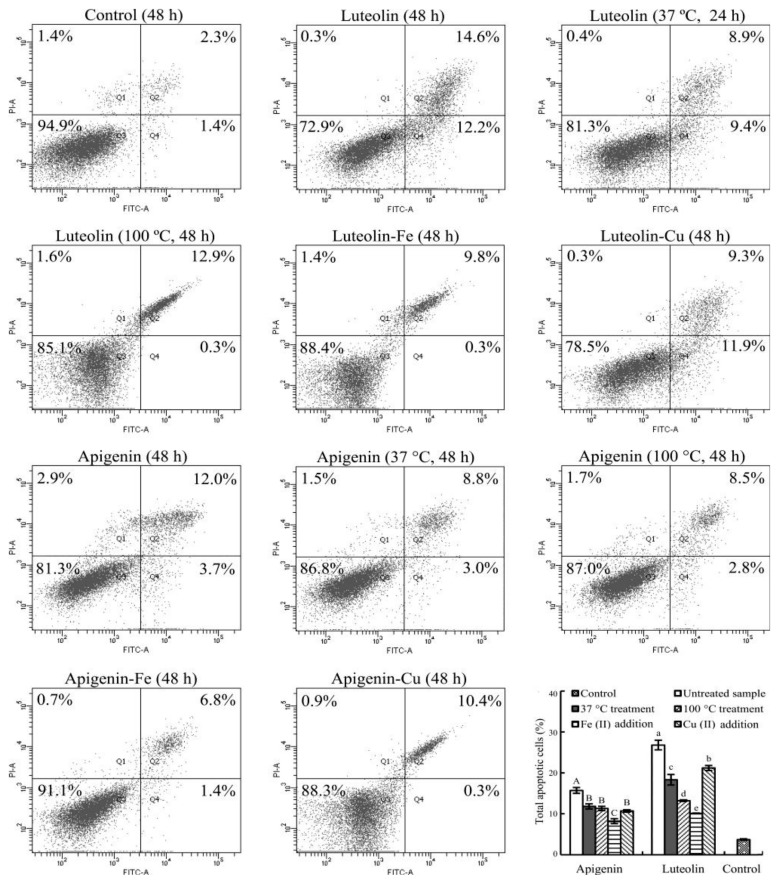
Cell percentages of the Hela cells treated with 0.1% DMSO (control) and 40 µmol/L flavone samples with or without thermal treatments and Fe/Cu addition for 48 h.

Apoptosis induction of the untreated and treated flavones were then assessed with the flow cytometry technique using the Annexin V-FITC/PI double staining and treatment times of 24 and 48 h (Figure 6 and Figure 7).

The control cells at 24 or 48 h only had total apoptotic cells (Q2 + Q4) of 3.4% or 3.7%. The cells treated with the untreated apigenin and luteolin for 24 (or 48) h led to increased total apoptotic cells about 12.8% and 16.1% (or 15.7% and 26.8%). If the cells were treated with the heated flavones, the total apoptotic cells were measured with the reduced percentages, especially using heat treatment at 100 °C. Subsequently, the total apoptotic cells were 7.3% and 10.2% (24 h) or 11.3% and 13.2% (48 h) with corresponding apigenin and luteolin treatments. When the two flavones were added with Fe^2+^, the respective apigenin and luteolin treatments resulted in the total apoptotic cells of 7.0% and 9.1% (24 h) or 8.2% and 10.1% (48 h). When the two flavones were added with Cu^2+^, the measured total apoptotic cells were 8.5% and 13.5% (24 h) or 10.7% and 21.2% (48 h) with respective apigenin and luteolin treatments. Data comparison further revealed how these treatments had positive or negative impacts on the apoptosis induction of the two flavones. Overall, the conducted heat treatment (especially at 100 °C) caused decreased total apoptotic cell proportions, while Fe addition also resulted in much decreased total apoptotic cell proportions than Cu addition did.

Further data analysis revealed that the measured ROS levels (Table 2) in the cells with a treatment time of 24 h were positively and significantly correlated with the detected total apoptotic cell percentages (Figure 6 and Figure 7), because the calculated Pearson’s correlation coefficient (i.e., *r*-value) of the two indices was 0.854 (*p* < 0.05). This correlation meant that the decreased abilities in ROS generation of apigenin and luteolin possibly resulted in decreased apoptosis induction. In other words, the used treatments brought about flavone degradation and lower abilities to generate ROS in Hela cells, and thereby led to decreased apoptosis induction. However, this phenomenon was no longer observed when the cells were treated with a longer time of 48 h. The treatment time of 48 h led to too much cell death or the lower number of viable cells (Figure 4). Consequently, only fewer viable cells in the media were able to generate ROS. This fact meant that much higher extent of apoptosis induction of apigenin and luteolin led to lower ROS generation. Therefore, the calculated Pearson’s correlation coefficient (*r*-value) of the two indices (i.e., ROS levels versus apoptotic cell percentages) decreased to 0.589 (*p* > 0.05). In this case, the measured apoptosis induction and ROS generation were positively but insignificantly correlated.

## 4. Discussion

Flavones, in general, have several −OH groups in their molecules, and; therefore, they as phenolic compounds are susceptible to oxidation. Heat treatment; thus, promotes flavone degradation, and is adverse to the stability and bio-activities of flavonoids. Polyphenols in the solid grape marc were degraded at 100–150 °C, leading to decreased anti-oxidation [29]. At the temperature of 250 °C, catechins might lose their DPPH radical scavenging ability completely due to the thermal degradation of catechins [30]. The anti-cancer activities of flavonoids (e.g., growth inhibition) are governed by their chemical structures [31,32]. Subsequently, structure changes of flavonoids will result in increased or decreased activity. It was found that heat treatment of cymaroside (i.e., luteolin-7-O-β-glucoside) led to the increased immuno-modulation by enhancing NK cells activity [33]. Additionally, the heated flavonoids showed decreased activities in the human colon carcinoma HCT-116 cells [19,20]. Thus, heat treatments (especially using 100 °C) in the present study caused greater degradation and decreased growth inhibition for both apigenin and luteolin.

It is well-known that Fe/Cu are capable of oxidizing flavonoids in solutions, resulting in flavonoid degradation [34]. However, flavonoids also can complex with multi-valent metal ions [35], resulting in changed stability. Thus, Fe/Cu added to apigenin and luteolin solutions might bring two major reactions: forming flavone–metal complexes and catalyzing flavone degradation [12,13]. From a chemical point of view, the redox cycling exists between transition metals and ligands [36]. Quercetin, rutin, and 3-hydroxyflavone in the presence of Fe^2+^/Cu^2+^ exhibited a significant decomposition, yielding semiquinone compounds [36]. Both apigenin and luteolin; thus, could be oxidized by the added Fe/Cu, resulting in changed chemical stability. However, apigenin and luteolin are different in their chemical structures that; thus, govern their stability changes in the presence of Fe^2+^/Cu^2+^. Normally, one luteolin molecule can chelate 1.5 Fe^2+^/Cu^2+^, but apigenin without two adjacent −OH groups is almost unable to chelate the two ions [11]. Apigenin in the present study; thus, was instable in the presence of Fe^2+^/Cu^2+^ (Table 1). On the contrary, luteolin has two adjacent −OH groups in its C-ring and thus can chelate the two ions; subsequently, it mainly showed enhanced stability in the presence of Fe^2+^/Cu^2+^ (Table 1). Moreover, the Cu-added luteolin also showed decreased stability at 37 °C (but not at 20 °C), which was attributed to the stronger oxidation of Cu^2+^ at this temperature. Consistent with the present finding, it was also found that quercetin bound with Fe^2+^ had inhibited oxidation, while that bound with Cu^2+^ received promoted oxidation [12]. In methanol medium, Cu^2+^ also promoted quercetin oxidation [13]. It was reasonable in the present study that the two flavones showed worse stability in the presence of Cu^2+^, especially at the higher temperature.

Hela cells have the potential to proliferate indefinitely and have been widely used for cancer research. It was reported that many flavonoids and their derivatives had the ability to inhibit Hela cells. Natural flavone eupatorine inhibited Hela cells through inducing cell-cycle arrest and apoptosis [37]. Wang and coauthors reported that quercetin could induce the apoptosis and autophagy of Hela cells [38]. Other researchers proved that quercetin had anti-cancer effects on HeLa cells via the adenosine 5‘-monophosphate -activated protein kinase (AMPK)-induced HSP70 and down-regulation of epidermal growth factor receptor (EGFR) [39]. In this study, Fe^2+^/Cu^2+^ showed different behaviors to affect the growth inhibition of apigenin and luteolin in the cells. Fe is one of the required nutritive elements for tumor growth [40], and is also reported to influence cell-cycle regulation at multiple sites [41]. Fe^2+^ chelation of flavonoids is one of the important mechanisms in response to their growth inhibition in cancer cells. Fe addition thereby decreased luteolin’s Fe-chelating activities, promoted apigenin oxidation, thus reasonably reduced its growth inhibition. Cu^2+^ is capable of inducing cellular oxidative stress, bringing DNA damage, and then initiating cell apoptosis [42]. Cu addition for the two flavones; thus, gave rise to two chemical reactions: enhancing flavone degradation and increasing cellular Cu content. The enhanced flavone degradation led to decreased growth inhibition, whilst the increased Cu content brought about extra oxidative stress or higher cytotoxic effect on the Hela cells. Subsequently, Cu addition of the two flavones in this study was observed with less decreased growth inhibition than Fe addition. The bio-activity changes of flavonoids in the presence of transition metal ions had been observed in other studies; for example, the complexes of rutin and dihydroquercetin with Fe, Cu, and Zn had higher anti-oxidation than the free counterparts as the inhibitors of asbestos-induced cell injury [43]. Similarly, the free radical scavenging ability of quercetin–Cu complex was higher than free quercetin [44]. Metal ions such as Cu, Fe, and Zn also had been evidenced to impact anti-microbial, anti-viral, and anti-inflammatory activities of flavonoids [45]. The present results also provided another evidence to show different effects of Fe^2+^/Cu^2+^ on anti-cancer activities of the two flavones.

Flavonoids have both anti- and pro-oxidation in cells, depending on flavonoid concentrations and free radical sources [46]. The pro-oxidation of flavonoids plays an important role in their anti-cancer activities, via promoting the generation of intracellular ROS in cancer cells [47]. In general, a relative higher flavonoid level in cancer cells leads to pro-oxidation, promotes ROS generation, and, thereby, induces DNA damage [48]. Pro-oxidation of a tea polyphenolic compound, epigallocatechin-3-gallate, has been proved to govern its growth inhibition on colorectal HT29 cells, oral squamous carcinoma SCC-25 and SCC-9 cells, and premalignant leukoplakia MSK-Leuk1cells [49], while cytotoxic effects of quercetin, morin, and kaempferol on promyelocytic leukemia HL-60 cells were found to be caused by their pro-oxidation [22]. Both heat treatment and Fe/Cu addition of apigenin and luteolin led to oxidation and, thereby, altered the redox potential of the two flavones; the assessed samples; thus, had different abilities to generate intracellular ROS, and then showed different growth inhibition on Hela cells. Moreover, the enhanced ROS generation in cells suggests cell apoptosis, because this phenomenon is regarded as a classic way to trigger cell apoptosis [50]. Thus, flavonoids such as quercetin, luteolin, chrysin, kaempferol, apigenin, myricetin, and baicalin showed clear apoptosis induction to the human esophageal adenocarcinoma OE33 cells and three human prostate cancer cells, resulting in increased total apoptotic cells [7,8]. The conducted treatments in this study; thus, decreased ROS generation and apoptosis induction of the two flavones in the cells. It is reasonable that decreased ROS generation of the two flavones with treatment time of 24 h was positively and significantly consistent with their decreased apoptosis induction, as the correlation analysis results showed.

## 5. Conclusions

Two flavones, apigenin and luteolin, in aqueous solutions, had degradation to different extents, while Fe^2+^/Cu^2+^ addition mainly resulted in stability (i.e., decreased degradation) for luteolin due to the formation of luteolin–metal complexes, but also led to instability (i.e., increased degradation) for apigenin. The flavone degradation was clearly enhanced at 37 °C (the classic temperature of cell culture) rather than 20 °C. The used heat treatments (37 and 100 °C) and Fe^2+^/Cu^2+^ addition were adverse to the anti-cancer activities of the two flavones against human cervical cancer Hela cells; subsequently, growth inhibition, DNA damage, and especially apoptosis induction (positively correlated with the intracellular ROS generation) of the two flavones were decreased. It is; thus, proposed that more attention should be paid to both heat treatment and some metal ions like Fe^2+^/Cu^2+^ due to their negative effects when assessing the bio-activities of flavonoid compounds. However, this study only aimed to verify how the used heating treatments and two metal ions impacted flavone stability and anti-cancer activities in vitro. The related molecular mechanisms and an in vivo investigation will be carried out in a further study.

## Figures and Tables

**Table 1:** Degradation rate constants (*k*, h^−1^) of apigenin and luteolin in solutions treated with two temperatures or added with Fe^2+^/Cu^2+^ s.

Indices		Added Metals (Flavones:Metals 3:1)	Apigenin	Luteolin
Temperature	37 °C	None	0.0207 ± 0.0012 ^F^	0.0214 ± 0.0004 ^c^
		Fe^2+^	0.0395 ± 0.0011 ^D^	0.0149 ± 0.0009 ^f^
		Cu^2+^	0.0480 ± 0.0015 ^C^	0.0176 ± 0.0021 ^e^
	100 °C	None	0.0226 ± 0.0001 ^E^	0.0245 ± 0.0006 ^b^
		Fe^2+^	0.0520 ± 0.0002 ^B^	0.0203 ± 0.0005 ^d^
		Cu^2+^	0.0728 ± 0.0010 ^A^	0.0317 ± 0.0004 ^a^
Significance	Temperature		**	**
	Metals		**	**
	Temperature × Metals		**	**

Different lowercase or capital letter superscripts after the values in the same column indicate that the means differ significantly according to one-way ANOVA (*p* < 0.05). The two asterisks indicate that the means differ significantly according to two-way ANOVA (*p* < 0.05).

**Table 2:** The measured reactive oxygen species (ROS) levels in the Hela cells treated with different samples for 24 and 48 h.

Flavones	Heat Treatment (°C)	Added Ions (Flavones:Metals 3:1)	ROS Levels (% of Control)	
			24 h	48 h
Apigenin	None	None	228.6 ± 2.4 ^A^	262.8 ± 1.5 ^A^
	37	None	211.7 ± 3.8 ^B^	260.0 ± 6.4 ^B^
	100	None	206.9 ± 3.4 ^C^	245.3 ± 1.6 ^C^
	None	Fe^2+^	205.6 ± 3.8 ^C^	223.6 ± 1.0 ^E^
	None	Cu^2+^	212.1 ± 1.6 ^B^	237.4 ± 1.9 ^D^
Luteolin	None	None	284.1 ± 8.2 ^a^	280.9 ± 3.8 ^a^
	37	None	271.8 ± 5.0 ^b^	262.9 ± 3.5 ^b^
	100	None	234.2 ± 7.7 ^c^	256.8 ± 2.5 ^c^
	None	Fe^2+^	232.1 ± 1.0 ^c^	225.1 ± 5.7 ^e^
	None	Cu^2+^	268.4 ± 2.7 ^b^	246.4 ± 0.7 ^d^

Different lowercase or capital letter superscripts after the values in the same column indicate that the means are significantly different according to one-way ANOVA (*p* < 0.05).
